# Anthropometric and physical characteristics in U16, U18 and U20 elite French youth rugby union players

**DOI:** 10.1371/journal.pone.0295623

**Published:** 2023-12-20

**Authors:** Alexis Peeters, Cedric Leduc, Julien Piscione, Mathieu Lacome, Christopher Carling, Nicolas Babault

**Affiliations:** 1 Sport Science Faculty, INSERM UMR1093-CAPS, University of Bourgogne Franche-Comte, Dijon, France; 2 Performance Department, French Rugby Federation (FFR), Marcoussis, France; 3 Institute for Sport, Physical Activity and Leisure, Carnegie Applied Rugby Research (CARR) Center, Carnegie School of Sport, Leeds Beckett University, Leeds, United Kingdom; 4 Sport Science and Medicine Department, Crystal Palace Football Club, London, United Kingdom; 5 University of Evry, University of Paris Saclay, Evry, France; 6 Performance and Analytics Department, Parma Calcio 1913, Parma, Italy; 7 FFF Research Center, Fédération Française de Football, Centre National du Football, Clairefontaine-en-Yvelines, France; 8 Laboratory Sport, Expertise and Performance (EA 7370), French Institute of Sport (INSEP), Paris, France; 9 Centre for Performance and Expertise, University of Bourgogne Franche-Comte, Sport Science Faculty, Dijon, France; Università degli Studi di Milano: Universita degli Studi di Milano, ITALY

## Abstract

The aims of this study in elite youth French players were to 1) describe the anthropometric and physical characteristics of international and non-international players from U16 to U20, and 2) compare these characteristics across age categories and playing standard (international or non-international). Altogether, 1423 players from the French Rugby Federation’s academies participated in a physical testing battery, part of its national young player development pathway. From seasons 2010 to 2020, players were assessed for anthropometric (body mass and height), off-field (bench press; isometric squat, vertical jump) and on-field physical characteristics (aerobic capacity: maximal aerobic speed [MAS]; speed: 10-m, 50-m sprint). A 2-way mixed model analysis of variance (ANOVA) was used to compare physical characteristics across age categories and playing standards. Two separate models were used for forwards and backs. A main statistical effect was observed for age category and playing standard (range p < 0.05 –p < 0.001). Pair-wise category comparisons showed that older players were generally taller, heavier, stronger, faster and demonstrated better aerobic qualities than younger peers. The same results were observed for INT compared with NI players while INT forwards were also taller and heavier than NI peers (range p < 0.01 –p < 0.001). Findings revealed a clear progression in anthropometric characteristics and physical qualities throughout the age development pathway in elite young French rugby players. Findings also identified certain physical qualities (strength, power and speed) necessary at younger levels to achieve international standard.

## Introduction

Rugby Union requires a range of physical skills in order to cope with the intermittent and combative demands of the sport [1]. As such, it is important for practitioners to evaluate the physical qualities of players. Evaluations also help assess the efficacy of conditioning programs, and in the youth game, support talent identification and development schemes [2,3]. To determine physical qualities, batteries of tests are systematically utilised by clubs and governing bodies [4,5]. Despite a lack of consensus regarding the specific protocols and technologies typically employed, results from testing generally show that strength, speed, power and aerobic capacity are key determinants to perform at elite standards [5,6]. In senior international Rugby Union, these physical qualities are notably correlated with key match-play performance indicators such as the frequency of and efficiency in actions such as tackling, collisions, ball carries, and rucks [7].

Clear differences in physical capacities have been highlighted across age categories and playing positions. Research by Darrall-Jones [8] showed that U18 and U21 players were taller, heavier and stronger than U16 peers. In contrast, no significant difference in either sprinting time (over distances of 5 m to 40 m) or aerobic capacity was observed across these three age categories. Peeters [9] showed that U18 players covered a greater total and high-speed distance (≥ 4 m.s^-1^) than U20 peers in international competition. Regarding the effect of playing position, forwards have been shown to be significantly taller, heavier, and stronger than backs [10]. In contrast, speed qualities were reportedly more developed among backs than forwards [10,11]. To our knowledge, however, no evidence is available on age-related physical performance in relation to playing position.

Differences in various physical qualities have also been observed across playing standards in rugby union. Match-play running activity in U18 academy players was substantially greater than that in U18 school-standard peers [12]. A study by Jones [13] showed that players from a professional regional academy had superior values for height, body mass, strength, 20 and 40 m sprint performance, and aerobic fitness, versus peers participating at school standards. These results suggest that the physical qualities underpinning performance are more developed in elite-standard youth players. To our knowledge however, no study has confirmed whether similar differences in physical characteristics also exist at higher standards of play and notably in national versus international players. Determining the physical qualities inherent to play at both standards could help governing bodies to evaluate and potentially optimise their talent selection and development criteria.

A limitation of the current body of research on anthropometric and physical qualities in elite youth rugby union is that studies conducted in international standard players are generally scarce. While U20 international play has received some attention [11,14], to our knowledge, no information is available on U18 international players. Similarly, a large proportion of existing studies have only examined populations from the southern hemisphere [11] or English club academy players [8,10,13]. As such, additional research on the physical qualities in youth players belonging to other rugby nations is warranted.

The aims of this study in young elite male French rugby union players were to 1) describe the anthropometric and physical qualities of international and non-international players from U16 to U20 and 2) identify potential differences in these qualities between age categories and playing standard (international or non-international status). Position-specific characteristics (backs versus forwards) were also investigated across these age categories and standards of play.

## Methods

### Experimental approach to the problem

A retrospective analysis from season 2010 to 2020 has been performed. In total, 1423 players in U16, U18 and U20 categories belonging to the French Rugby Federation’s national academies took part in the national fitness testing battery which is part of the national development pathway. All players were assessed in a range of anthropometric (body mass and height), off-field (bench press; isometric squat, vertical jump) and on-field measures (aerobic capacity: MAS; speed: 10-, 50-m sprint). International status was applied to players who had played at least one official match for the U20 French national team or the senior French national team while the remaining players were classed as non-international (NI). A further distinction has been considered between forwards (players’ jersey numbers from 1 to 8) and backs (numbers 9 to 15).

### Subjects

**Fig 1:**
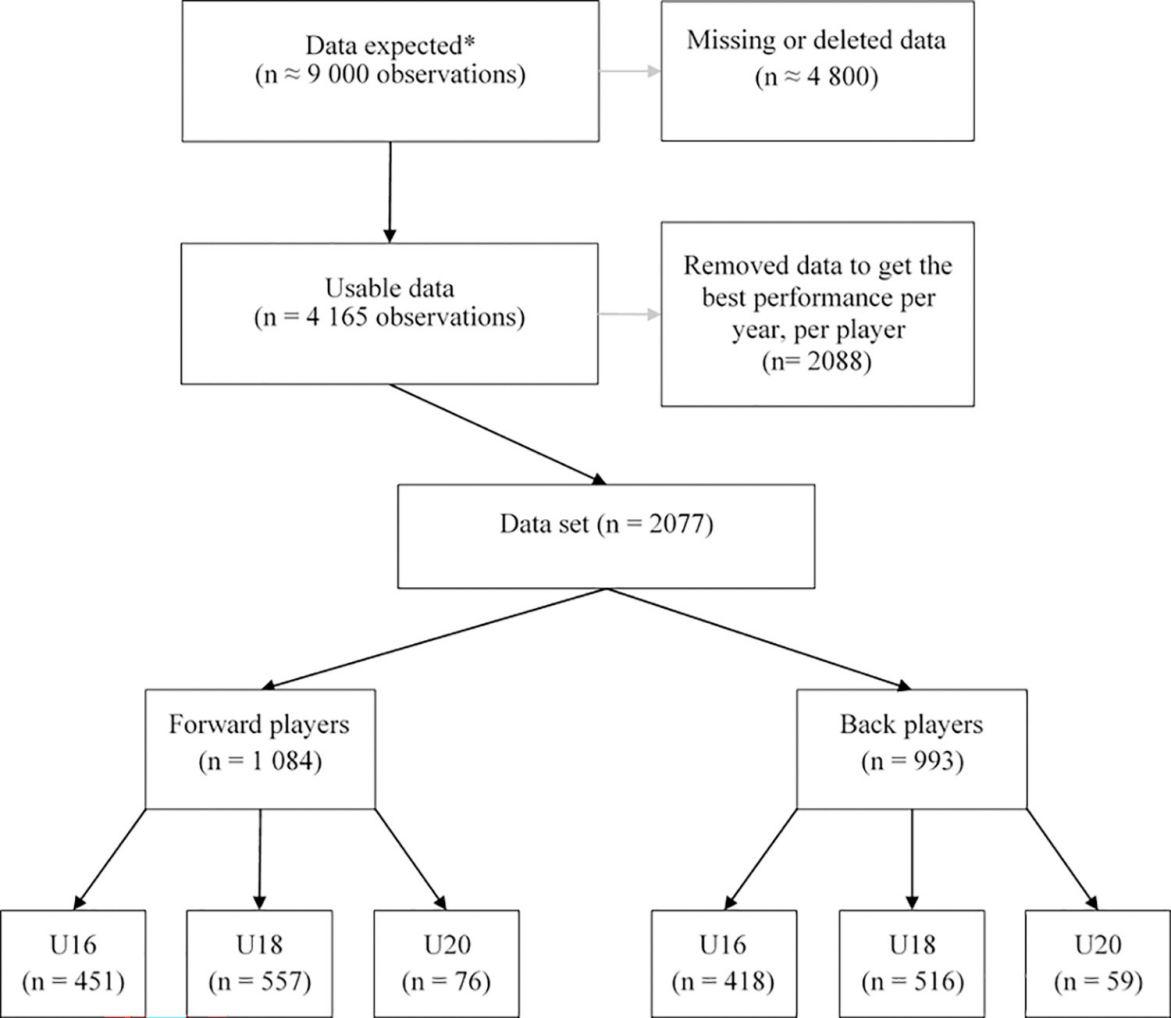
Flow chart diagram regarding participant’s inclusion. * This number has been extrapolated based on the number of players per academy tested each year.

The inclusion process is illustrated in Fig 1. A total of 1423 male elite rugby union players in U16, U18 and U20 categories were included from seasons 2010–2011 to 2019–2020 consecutively. The dataset contained results from certain players who were tested in all three age categories over the course of their international pathway. Players trained in the French Rugby Federation’s national academies approximately 7 hours per week on the field and 5 hours in the gym (specific strength and conditioning sessions) respectively. All test-related data were routinely collected as part of the French Rugby Federation’s national player development pathway. Nevertheless, all participants provided informed consent prior to starting the study. A parent or guardian provided written consent for all players under 18 years old. Approval for the study was granted by the French Rugby Federation’s science and research commission and the recommendations of the Declaration of Helsinki were respected.

### Procedures

**Fig 2:**
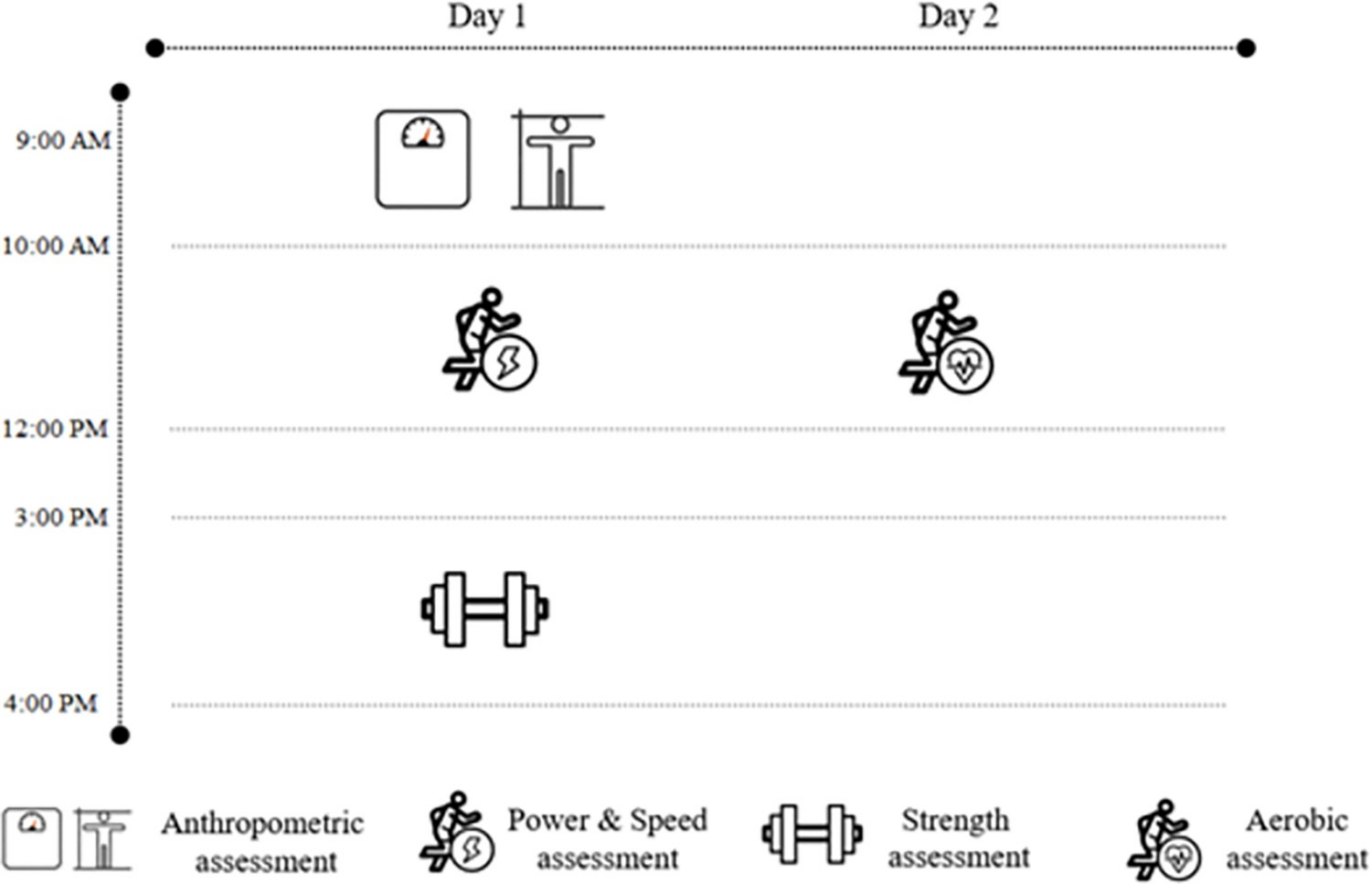
Schematic representation of the study design.

The present data were collected over a ten-year period in all ten of the French Rugby Federation’s national rugby academies. Three test session periods were identified for each year: pre-season (i.e. September to December), in-season (i.e. February to April) and end-season (i.e. June to July). All players were assessed for a range of anthropometric (body mass and height) and physical (strength: bench press; isometric squat; power: vertical jump; aerobic capacity: maximal aerobic speed (MAS); speed: 10-, 50-m sprint) characteristics. To reduce the possible influence of fatigue, testing sessions were spread over two days (Fig 2). On day 1, anthropometric characteristics, power and speed were tested in the morning while strength assessments were performed in the afternoon. The aerobic assessment was conducted in the morning of day 2. Tests were fully explained and demonstrated prior to each assessment by the strength and conditioning coach in charge of the testing battery. All procedures are further described in the methodology section. Only the player’s best performance over the course of the season was retained for each physical test.

### Anthropometric characteristics

Each player was weighed barefoot and wearing only their underwear. Previous research reported a typical error of measurement (TEM) expressed as % coefficient of variation (CV) of 0.6% [11]. Standing height was recorded with the player barefoot, and his arms positioned at his sides. His heels, buttocks, upper back, and head were in contact with a wall. The measurement was recorded as the height from the floor to the vertex of the head with a tape measure. Previous research has reported a TEM % CV of 0.2%, [11].

### Upper body strength

The 3-repetition maximum (RM) bench press was used to assess upper-body strength [8,15]. The assessment commenced with a standardised warm-up protocol, including progressively increasing load, at submaximal and near 3RM loads. Players had 5 attempts to reach their 3RM performance, with 3 to 5 minutes recovery between sets. The coaching staff controlled the execution: the head, upper back and buttocks were in contact with the bench, and foot in contact with the floor or raised with a wedge.

### Lower body strength

Players’ lower-body strength was assessed using a portable force plate (Kistler 9286B, Kistler, Winterthur, Switzerland) during an isometric squat. Force plate data were sampled at 1000 Hz using Bioware version 5.2 (Kistler, Winterthur, Switzerland). Knee angulation was set at 90 degree and controlled with a manual goniometer. Following a standardised warm-up, players had 2 attempts (3 to 5 minutes recovery between sets) with the best values conserved for analysis. Peak isometric force in kg (with body mass, measured at the beginning, removed) was analysed. Previous research has reported good reliability for the Kistler 9286B system with a CV < 3% and an intraclass correlation coefficient (ICC) > 0.95 for force output assessment [16].

### Lower body power

The countermovement jump (CMJ) was used to assess lower-body power output. Jump height in centimetres (cm) was measured using the Optojump photocell system (Microgate, Bolzano, Italy). After a standardized warm-up, participants completed a set of 4 repetitions of CMJ, with 3 to 5 seconds recovery between jumps. They were asked to jump as high as possible while keeping their back straight and respecting the reference marks on the ground to avoid any movement either forwards or backwards. The mean value of the 3 best jump heights was used ([17]). Previous testing of the Optojump system has demonstrated excellent reliability, with ICC = 0.99, low CV (2.2%), and low typical error (±2.43 cm) for CMJ height assessment [18].

### Speed

Sprint times were assessed during two 10-m sprints and two 50-m sprints using electronic timing gates (Smart Speed, Fusion Sport, Australia) with a 0.01-second accuracy. The speed session assessment started with a standardised warm-up protocol. Players commenced their sprint from a standing position with their feet set 0.5 m behind the first timing gate [19]. 10-m sprint trials were separated with 1-min recovery time, and 3-min recovery for 50-m sprints. The best of the two times was selected for analysis. Previous research has reported intraclass correlation coefficients and CVs for 10-m, and 40-m sprint times of ICC r = 0.94 and CV = 1.4%, and ICC r = 0.96 and CV = 1.2%, respectively [8].

### Aerobic capacity

The Bordeaux 2 University Test (TUB 2) was performed to determine maximal aerobic speed (MAS) [20]. This is an intermittent progressive running test adapted from the Leger and Boucher test [21], consisting of bouts of 3 min interspersed with 1 min passive rest intervals. Velocity was increased by 2 km.h^-1^ from 8 to 12 km.h^-1^ and then by 1 km.h^-1^ until voluntary exhaustion. The test was performed on a tartan outdoor track. Players were all fully familiarised with the test procedure. The MAS score corresponded to the speed reached by the participant during the last 3 min bout. Comparative testing in athletes has shown very similar values for MAS generated using the Leger and Boucher test (17.2 ± 1.1 km.h^-1^) and the TUB 2 (17.4 ± 1.0 km.h^-1^) respectively (p>0.05) [22].

### Statistical analysis

Players were categorized by playing position (forwards and backs), age category (U16, U18 and U20) and playing standard (non-international or international status). For each age category, international status was applied to players who had played at least one official match for the U20 French national team or the senior French national team. This designation was arbitrary and chosen by the present research team as we deemed these two categories to be the best representation of French rugby at international standards. Statistical analyses were conducted using the JASP software (Ver 0.14, JASP Team, 2020, University of Amsterdam, Amsterdam, The Netherlands), after anonymising data. The Kolmogorov-Smirnov normality test was performed to verify the distribution of each variable included in the analysis. A 2-way mixed model analysis of variance (ANOVA) was used to investigate physical characteristics differences between age categories and playing standard status. Two separate models were used for forwards and backs. When a significant difference (p < 0.05) was observed, Bonferroni post hoc analyses was used to detect pairwise differences. Partial eta squared (pη^2^) was calculated for each significant pairwise comparison to obtain the magnitude of the difference. Values of 0.01, 0.06, and above 0.14 were considered to represent small, medium and large differences, respectively [23].

## Results

Results from the ANOVA test are presented in Table 1 for both positions and Tables 2 and 3 presents anthropometric and physical characteristics for forward and back players respectively in addition to the results of the post-hoc tests.

**Table 1:** Results from the mixed-model ANOVA for both positions.

		Forwards			Backs		
		F	p	p η^2^	F	p	p η^2^
Height	Age category	3.74	0.02 *	0.01	8.48	< .001 ***	0.02
	Status	12.76	< .001 ***	0.01	1.12	0.29	0
	Age category ✻ Status	0.72	0.49	0,00	0.51	0.6	0
Body mass	Age category	27.39	< .001 ***	0.05	59.73	< .001 ***	0.11
	Status	8.36	0.004 **	0.01	1.94	0.16	0
	Age category ✻ Status	0.92	0.40	0,00	2.23	0.11	0.01
Bench Press	Age category	176.86	< .001 ***	0.28	204.69	< .001 ***	0.33
	Status	9.22	0.002 **	0.01	19.74	< .001 ***	0.02
	Age category ✻ Status	3.64	0.03 *	0.01	3.29	0.038 *	0.01
Isometric Squat	Age category	67.31	< .001 ***	0.17	55.3	< .001 ***	0.16
	Status	17.32	< .001 ***	0.03	17.64	< .001 ***	0.03
	Age category ✻ Status	2.79	0.06	0.01	2.28	0.10	0.01
CMJ	Age category	41.75	< .001 ***	0.09	27.06	< .001 ***	0.07
	Status	6.27	0.012 *	0.01	10.83	0.001 **	0.01
	Age category ✻ Status	1.43	0.24	0,00	0.74	0.48	0
Sprint 10-m	Age category	12.17	< .001 ***	0.03	5.58	0.004 **	0.01
	Status	8.08	0.005 **	0.01	22.34	< .001 ***	0.03
	Age category ✻ Status	0.59	0.55	0,00	0.17	0.84	0
Sprint 50-m	Age category	18.54	< .001 ***	0.04	15.39	< .001 ***	0.04
	Status	3.29	0.07	0,00	16.68	< .001 ***	0.02
	Age category ✻ Status	1.31	0.27	0,00	0.38	0.68	0
MAS	Age category	8.95	< .001 ***	0.03	3.85	0.02 *	0.01
	Status	0.59	0.44	0,00	9.64	0.002 **	0.02
	Age category ✻ Status	0.21	0.81	0,00	2.71	0.07	0.01

*: p < 0.05

**: p < 0.01

***: p < 0.001.

**Table 2:** Anthropometric and physical characteristics of international and non-international forward players by age categories.

Forwards	U16		U18		U20		Significant Post Hoc differences
	NI	I	NI	I	NI	I	
Observations (n)	403	48	455	102	27	49	
Height (cm)	184.2 ± 6.6	187.3 ± 7.2	186.4 ± 6.9	188.2 ± 6.7	186.5 ± 9.3	190.1 ± 7.4	INT > NI
Body mass (kg)	92.9 ± 12.5	94.8 ± 10.3	98.9 ± 11.9	103.8 ± 9.3	101.9 ± 9.6	106.1 ± 10.7	U16 < U18, U20; INT > NI
Bench press (kg)	78.3 ± 13	79.1 ± 13.3	100.2 ± 16.5	110 ± 16.7	118.2 ± 19.9	124.4 ± 18.6	U16 < U18, U20; U18 < U20; INT > NI; &*
Isometric Squat (kg)	140.1 ± 28.5	146.7 ± 29.7	164.6 ± 33.5	188.4 ± 38.6	191.8 ± 38.3	211.4 ± 38.5	U16 < U18, U20; U18 < U20; INT > NI
CMJ (cm)	31.2 ± 5.4	31.4 ± 4.3	33.9 ± 5.1	35.7 ± 3.9	37 ± 5.2	39.3 ± 5.4	U16 < U18, U20; U18 < U20; INT > NI
MAS (km.h^-1^)	15.2 ± 1.4	15.1 ± 1.3	15.8 ± 1.3	15.7 ± 1.3	15.9 ± 0.9	15.6 ± 1.3	U16 < U18
Sprint 10-m (s)	1.82 ± 0.1	1.79 ± 0.09	1.8 ± 0.09	1.76 ± 0.07	1.75 ± 0.08	1.73 ± 0.07	U16 > U18, U20; U18 > U20; INT < NI
Sprint 50-m (s)	7.01 ± 0.41	6.99 ± 0.36	6.87 ± 0.37	6.71 ± 0.28	6.71 ± 0.36	6.64 ± 0.3	U16 > U18, U20

NI: non-international; I: international; CMJ: countermovement jump; MAS: maximal aerobic speed.

*&: U16 INT < U18 INT, U18NI, U20 INT, U20 NI; U18 INT < U20 INT; U18 INT > U16 NI, U18 NI; U20 INT > U16 NI, U18 NI; U16 NI < U18 NI, U20 NI; U18 NI < U20 NI.

**Table 3:** Anthropometric and physical characteristics of international and non-international back players by age categories.

Backs	U16		U18		U20		Significant Post Hoc differences
	NI	I	NI	I	NI	I	
Observations (n)	377	41	438	78	32	27	
Height (cm)	176.6 ± 6.3	176.9 ± 6.4	178.3 ± 6.4	179.9 ± 6	180.4 ± 6.5	181 ± 5.5	U16 < U18, U20
Body mass (kg)	72.3 ± 7.6	73.1 ± 7.9	77.8 ± 8.2	81.4 ± 8.4	85.9 ± 7.1	85.5 ± 6.7	U16 < U18, U20; U18 < U20
Bench Press (kg)	72.4 ± 10.8	75.6 ± 10.5	91 ± 12.9	101 ± 12.9	108.4 ± 11.5	116.7 ± 11.7	U16 < U18, U20; U18 < U20; INT > NI; &*
Isometric Squat (kg)	134.5 ± 25.5	140.2 ± 27.8	157 ± 29.7	173.1 ± 27.4	173.6 ± 22.6	200.2 ± 30.5	U16 < U18, U20 ; U18 < U20 ; INT > NI
CMJ (cm)	36.9 ± 4.5	38 ± 4.3	39.4 ± 4.9	41.7 ± 4.4	41.4 ± 4.4	43.5 ± 5.6	U16 < U18, U20 ; U18 < U20 ; INT > NI
MAS (km.h^-1^)	16.7 ± 1	16.9 ± 0.9	17 ± 1.1	17.1 ± 1	15.7 ± 0.6	17.2 ± 0.8	INT > NI
Sprint 10-m (s)	1.73 ± 0.08	1.69 ± 0.07	1.71 ± 0.07	1.67 ± 0.07	1.7 ± 0.07	1.65 ± 0.06	U16 > U18, U20 ; INT < NI
Sprint 50-m (s)	6.53 ± 0.27	6.42 ± 0.21	6.42 ± 0.25	6.26 ± 0.19	6.38 ± 0.23	6.23 ± 0.16	U16 > U18, U20 ; INT < NI

NI: non-international; I: international; CMJ: countermovement jump; MAS: maximal aerobic speed.

*&: U16 INT < U18 INT, U18NI, U20 INT, U20 NI; U18 INT < U20 INT; U18 INT > U16 NI, U18 NI; U20 INT > U16 NI, U18 NI; U16 NI < U18 NI, U20 NI; U18 NI < U20 NI.

### Anthropometric characteristics

The analysis of forward players’ anthropometric characteristics revealed a significant effect of age category and status on both height (p < 0.05 and p < 0.001, respectively) and body mass (p < 0.001 and p < 0.01, respectively). Significant differences were reported between NI and INT forwards for height (+2.9 ± 1.6 cm, p < 0.001) and body mass (+3.7 ± 2.5 kg, p < 0.01). Significant differences were reported between U16 and U18, and U20 forwards for body mass (-7.5 ± 2.6 kg, p < 0.001, -10.2 ± 4.3 kg, p < 0.001, respectively).

The analysis of back players’ anthropometric characteristics revealed a significant effect of age category for both height (p < 0.001) and body mass (p < 0.001). Significant differences were reported between U16 and U18, and U20 backs for height (-2.3 ± 1.6 cm, p < 0.01, and -3.9 ± 2.6 cm, p < 0.01, respectively) and body mass (-6.8 ± 1.9 kg, p < 0.001, and -13.0 ± 3.0 kg, p < 0.001, respectively). A significant difference was also reported between U18 and U20 backs for body mass (-6.1 ± 3.0 kg, p < 0.001).

### Off-field testing

The analysis of strength and power characteristics revealed a significant effect of age category and status, in both playing positions (p < 0.05 –p < 0.001) for the bench press test, isometric squat and CMJ height. In both positions, significant differences were observed between INT and NI players for the bench press (forwards: +5.6 ± 3.6 kg, p < 0.01, backs: +7.2 ± 3.2 kg, p < 0.001), isometric squat (+16.6 ± 7.8 kg, p < 0.001, and +16.1 ± 7.5 kg, p < 0.001, respectively) and CMJ height (1.4 ± 1.1 cm, p < 0.05, and 1.8 ± 1.1 cm, p < 0.01, respectively).

Significant differences were reported between U16 and U18, and U20 forwards and between U18 and U20 forwards, for upper- body strength (U16 vs U18: -26.4 ± 3.9 kg, p < 0.001, U16 vs U20: -42.6 ± 6.1 kg, p < 0.001, and U18 vs U20: -16.2 ± 5.6 kg, p < 0.001) and lower-body strength (-33.1 ± 8.6 kg, p < 0.001, -58.2 ± 13.2 kg, p < 0.001, and -25.1 ± 12.3 kg, p < 0.001, respectively) and lower-body power (-3.5 ± 1.2 cm, p < 0.001, -6.9 ± 1.9 cm, p < 0.001, and -3.3 ± 1.7 cm, p < 0.001, respectively).

Values for upper- body strength significantly differed between U16 and U18, and U20 backs and between U18 and U20 backs, for (U16 vs U18: -22.0 ± 3.1 kg, p < 0.001, U16 vs U20: -38.5 ± 5.4 kg, p < 0.001, and U18 vs U20: -16.5 ± 5.1 kg, p < 0.001) and lower-body strength (-27.6 ± 7.7 kg, p < 0.001, -49.5 ± 12.8 kg, p < 0.001, and -21.9 ± 12.0 kg, p < 0.001, respectively) and lower-body power (-3.1 ± 1.2 cm, p < 0.001, -5.0 ± 1.8 cm, p < 0.001, and -1.9 ± 1.7 cm, p < 0.05, respectively).

The mixed model ANOVA revealed a significant category x status interaction for the bench press in both positions. The post-hoc analysis revealed differences between U16 INT and U18 INT and U20 NI, and U18 INT and U18 NI, and U20 INT for forwards and backs (Tables 2 and 3, respectively). Post-hoc analyses revealed no significant difference in U16 INT versus U16 NI and U20 INT versus U20 NI.

### On-field testing

The analysis of aerobic capacities revealed a significant category effect in both playing positions (p < 0.01 –p < 0.001), and a status effect but only for back players. Post-hoc test indicated a significant difference between INT and NI backs (+0.6 ± 0.4 km.h^-1^, p < 0.01), and between U16 and U18 forwards (-0.6 ± 0.3 km.h^-1^, p < 0.001).

The analysis of 10 m sprint time revealed a significant effect of category and status in both groups (p < 0.05 –p < 0.001). Post-hoc test indicated significant differences between INT and NI players in forward and back positions (-0.03 ± 0.02 s, p < 0.01, and -0.04 ± 0.02 s, p < 0.001, respectively). Significant differences were observed between U16 and U18, and U20 forwards, and between U18 and U20 forwards for 10 m sprint time (U16 vs U18: +0.03 ± 0.02 s, p < 0.01, U16 vs U20: +0.07 ± 0.03 s, p < 0.001, and U18 vs U20: +0.04 ± 0.03 s, p < 0.01). Analysis of Back’s 10 m sprint times revealed significant differences between U16 and U18 and U20 (+0.02 ± 0.02 s, p < 0.05, and +0.04 ± 0.03 s, p < 0.05).

The analysis of 50 m sprint times revealed a significant effect of category in both positions (p < 0.001) and status only for backs (p < 0.001). Post-hoc test indicated a significant difference between INT and NI backs (-0.14 ± 0.07 s, p < 0.001). Significant differences were observed between U16 and U18, and U20 forwards for 50 m sprint times (+0.21 ± 0.10 s, p < 0.001, and +0.33 ± 0.15 s, p < 0.001). Analysis 50 m sprint times in backs revealed significant differences between U16 and U18, and U20 (+0.14 ± 0.06 s, p < 0.001, and +0.18 ± 0.11 s, p < 0.001).

## Discussion

The main aim of this study was to compare anthropometric (height and body mass) and physical characteristics (strength, power, speed, aerobic capacities) in French Elite rugby union players across three age categories (i.e., under 16s, under 18s and under 20s) and two playing positions (forwards and backs). A second major aim was to compare these characteristics in international versus non-international standard players. Results showed that an age effect was almost systematically observed, with this effect also observed in both playing positions. In U16, U18 and U20 pair-wise comparisons, older players were generally taller, heavier, stronger, faster and demonstrated better aerobic qualities than younger peers. Results also highlighted that INT players were faster, stronger, more powerful, and demonstrated better aerobic capacities than NI players while INT forwards were also taller and heavier than NI peers.

### Anthropometric characteristics

The present study demonstrated a significant difference in height and body mass across age categories (U16, U18 and U20) and in both playing positions within these. Logically, older players were generally taller and heavier than younger peers. Indeed, research conducted in English academy players observed a similar pattern with an increase in anthropometric characteristics between U16, U18 and U21 categories [8]. This is likely explained by the trajectory of growth and maturation following peak height velocity which is further influenced by large increases in testosterone during this period [24]. Strengthening this suggestion regarding the effects of maturation, greater changes in anthropometric characteristics were notably observed between players from U16 to U18 than from U18 to U20 categories, with this pattern confirmed for both playing positions.

To our knowledge, the present study is the first to compare anthropometric characteristics between young INT and NI rugby union players. The comparison between playing standards showed that INT players were heavier than NI players, and INT forwards were taller than NI peers. A related study revealed that academy players were taller and heavier than school players [13]. In other sports such as soccer, comparable results have been observed between international, professional and amateur players [25,26]. Researchers demonstrated that body size was an important selection criterion, especially when comparing international and amateur players. These similarities across studies are not surprising considering the potential on-pitch advantages induced by anthropometric and physical qualities in team sports [25].

Similar findings have been observed in the characteristics of professional versus non-professional rugby union players, although unlike the current study, the authors did not consider the potential effect of playing position [27]. Indeed, here, height and body mass were differentiating factors in INT forward players. Considering the high contact loads that forwards face in elite-standard rugby union [28], and the greater focus on strength and power development in these positions, these differences can be expected. Consequently, monitoring and developing body mass among forward players specifically, would seem to be of particular importance for elite national talent identification and development pathways while helping to create norms across age categories. In contrast, the same result was not observed among the present back players suggesting that anthropometric qualities might not be discriminating factors for this specific position in younger age categories.

### Off-field testing

The results for the U16 and U20 cohorts presented similar or greater values for upper and lower body strength to those reported in U16 and U21 national team players in New Zealand [27]. The authors also reported similar trends to the present study with older players tending to be stronger than their younger counterparts [27]. Similarly, differences between age categories were observed in English academies between U16, U18 and U21 players [8]. In the present cohort, significant differences were also observed for upper and lower body strength and power in both playing positions, with players becoming stronger and more powerful with age. Taking into consideration the aforementioned maturation changes as well as the increase in training workloads common to older age categories [29], it is not surprising to observe these trends across the current and previous investigations.

To our knowledge, this study is the first to highlight differences in strength and power characteristics between INT and NI players. Previous research by Jones [13] focused on identifying differences in players at lower standards of play (e.g. school vs. academy). The significant statistical interaction between player age and playing standard status regarding upper body strength suggests that this might be a key indicator when tracking changes in physical performance across a national player development pathway. It is not surprising that values for lower body strength and power were lower amongst NI players suggesting the importance of lower body conditioning for competing at INT youth standards. These differences between INT and NI players can be linked to a myriad of intrinsic (i.e. genetic, training background etc…) and extrinsic factors (e.g. individual development, club policies etc…). Nevertheless, we can suppose that particular attention is paid to INT players by clubs and the present rugby federation to load monitoring and individualized training prescriptions to aid development of future international players. The observation that INT players were stronger than those at national standards is noteworthy and is of importance to both governing bodies as regards player identification and development in the highest-level pathway and to coaching staff for their national team selection policies.

### On-field testing

In comparison to the aforementioned physical qualities, no statistical difference was observed regarding MAS values either between playing standards or across age categories despite the tendency for values to increase with age in the international players. This second finding is comparable to findings reported by Darrall-Jones [30], showing that physical fitness in professional rugby club players remained fairly stable from U16 to senior categories. The authors suggested that the players’ ability to perform high intensity running nonetheless increased with age, although this was not directly translated into greater performance notably due to the detrimental effect of body mass on change of direction. Despite differences in the test protocols employed (i.e., continuous test, no changes in direction), we can suggest that age-related increases in body mass could also have had a similar detrimental effect on the present players’ MAS performance owing to the increased energetic cost linked to the incremental changes in speed during the test [30]. As such, practitioners working with young rugby players must account for body mass changes when interpreting data on aerobic fitness capacities across a range of ages as well as the protocol utilised.

Previous research has shown that sprint performance in U16 rugby players notably can discriminate career progression to the highest level [31]. However, a recent meta-analysis [5] revealed no differences in ≤10-m sprint time from the U16 to U20 categories across different standards (school to national). Here, running speed over short distances (i.e., 10m) significantly differed in relation to age and playing standard generally, and for both playing positions. This result implies that the present young players not only become quicker over time, but that those who achieved international standards were faster. This finding is noteworthy as linear running speed is associated with meters made, evasion and line and tackle breaks in high-standard rugby union match-play [5,32]. Also of note is the plateau observed between U18 and U20 forward and back players over longer distances (from ≥10 to 50m). Those results partly align with the conclusions of the aforementioned recent meta-analysis which showed no clear difference over longer sprint distances in rugby union players across different age categories for all playing positions [5]. The present results also showed that INT backs were faster than NI players. These results contradict those reported by Barr [14], where sprint performance did not improve from U20 international to senior international standards.

Collectively the aforementioned results, associated with the reported greater lower body strength in senior professional players compared with U20 peers [27], suggest that practitioners must focus on achieving the transfer of strength qualities to sprinting while respecting key technical components to improve sprinting performance. As a result of growth, attention must be paid throughout the youth player’s journey as the development of longer limbs might influence stride length and frequency enhancing sprint performance at a young age (< 16 years) [5] which is then followed by a period of significant weight increase (> 16 years) where the opportunity for developing speed qualities is reduced [5]. Additionally, the impact of body mass on speed development merits discussion. Casserly [33] suggested that small increases in body mass act as a mediator of speed development from U18 to U20 categories, explaining the plateau observed in timing speed over longer distances. Therefore, performance over shorter speed distances (< 10-m sprint) might arguably be considered a more pertinent indicator to help identify future potential international players.

### Limitations

This study has several limitations. First, retrospective data from a national testing battery implemented by a governing body were utilised thus the number and choice of tests was restricted and potentially limits the impact of the findings. For example, only body mass was used while information related to body composition would arguably have provided further insights into the differences observed between groups. Second, the current investigation did not include any information related to senior INT or NI players. As such, it remains to be seen how the physical qualities of the present young French players evolve towards senior playing standards (international or national). Knowledge of this progression would help the governing body to chart the overall physical profile landscape of its players and possibly forecast future international players both more accurately and sooner. Third, limited data were available in U20 players (n<100). This can be explained by a lack of access to NI players within this age group, as they were often integrated into the professional group and not selected for duty with the U20 national team. Finally, future studies could include information on training load dose responses to help further knowledge and explain the differences and evolutions observed across age categories and status.

## Conclusions

The aim of the current study was to assess and compare anthropometric and physical characteristics between age categories and status. The main findings generally revealed a clear progression in certain anthropometric characteristics and physical qualities throughout the development pathway of young French players and their progress to attaining international standards. This highlights the efficiency of the present pathway in helping players become notably stronger and faster over time. While the testing battery can help identify future young international players, future studies using additional physical test measures and training load dose responses would help improve understanding of the present results.

### Practical applications

- Differences in anthropometric characteristic were observed with regard to player age and status. Consequently, governing bodies could consider anthropometric characteristics as initial and simple indicators to track forward players notably within their pathway.
- Regarding off-field testing, measures of upper body strength assessed through the bench press highlighted differences between age categories and status. Therefore, this test could also be used longitudinally to help identify, select and develop potential INT players.
- Testing sprinting ability over a shorter distance (>10 m) seems most appropriate to distinguish physical performance between INT and NI players. In contrast, measuring speed over longer distances, such as 50 m, seems to be more adapted to back players. Specific attention needs to be given to acceleration and maximal speed qualities when it comes to talent identification and development.

## Supporting information

S1 Checklist STROBE statement—Checklist of items that should be included in reports of observational studies. (DOCX)
